# The trend of the distribution of ectopic pregnancy sites and the clinical characteristics of caesarean scar pregnancy

**DOI:** 10.1186/s12978-022-01472-0

**Published:** 2022-08-20

**Authors:** Panpan Tang, Xiaomao Li, Wenwei Li, Yunhui Li, Yu Zhang, Yuebo Yang

**Affiliations:** Gynecology Department, The Third Affiliated Hospital of Sun Yat-Sen University, Guangzhou, China

**Keywords:** Ectopic pregnancy, Distribution, Trend, Tubal pregnancy, Caesarean scar pregnancy

## Abstract

**Background:**

Ectopic pregnancy is a life-threatening occurrence and is an important cause of pregnancy-related mortality. We launched the study to investigate the distribution and its variation trend of the ectopic pregnancy sites and the clinical characteristics of caesarean scar pregnancy, to provide information for further clinical practice.

**Methods:**

A total of 3915 patients were included in our study to calculate the distribution of the implantation sites of ectopic pregnancies. Then, we performed a χ^2^ test for trend and calculated the quantity of each type of ectopic pregnancy during 2012–2015 and 2016–2019 to analyse the variation trend.

**Results:**

(1) The proportion of each site of ectopic pregnancy was as follows: tubal pregnancy (84.70%), ovarian pregnancy (1.56%), caesarean scar pregnancy (8.63%), abdominal pregnancy (0.61%), cornual pregnancy (2.68%), cervical pregnancy (0.49%), heterotopic pregnancy (0.43%). (2) Through the χ^2^ test for trend, the ratio of caesarean scar pregnancy to ectopic pregnancy showed an upward trend (*P* = 0.005). From 2012 to 2015 and 2016–2019, the ratio of caesarean scar pregnancy to ectopic pregnancy increased from 5.74 to 11.81% (*P* < 0.001). (3) A total of 72.78% (246/338) caesarean scar pregnancy patients had one caesarean delivery, 25.15% (85/338) had two caesarean deliveries, and 2.07% (7/338) had three caesarean deliveries. A total of 80.18% (271/338) had aborted before. The most common clinical manifestations were amenorrhea (98.52%), abdominal pain (25.74%) and vaginal bleeding (67.76%), the most common sign was uterine enlargement (46.75%).

**Conclusion:**

As the ratio of caesarean scar pregnancy increases, the caesarean delivery rate should be decreased to decrease the morbidity of caesarean scar pregnancy.

## Plain language summary

Ectopic pregnancy occurs when a fertilized ovum implants outside the endometrium of the uterine cavity, which is a life-threatening occurrence and is an important cause of pregnancy-related mortality. With the increase in pelvic and intrauterine operations, the distribution of ectopic sites has been changing, but the variation has been insufficiently studied. To investigate the distribution of ectopic sites and its variation trend in depth, we collected the data of 3915 ectopic pregnancy cases from the third affiliated hospital of Sun Yat-Sen University.

Through χ^2^ test for trend, the ratio of caesarean scar pregnancy to ectopic pregnancy showed an upward trend (*P* = 0.005). From 2012 to 2015 and 2016–2019, the ratio of caesarean scar pregnancy to ectopic pregnancy increased from 5.74 to 11.81% (*P* < 0.001). As the increasing of the ratio of caesarean scar pregnancy to ectopic pregnancy, the clinical characteristics of caesarean scar pregnancy was analysed.

A total of 72.78% (246/338) caesarean scar pregnancy patients had one caesarean delivery, 25.15% (85/338) had two caesarean deliveries, and 2.07% (7/338) had three caesarean deliveries. A total of 80.18% (271/338) had aborted before. The most common clinical manifestations were amenorrhea (98.52%), abdominal pain (25.74%) and vaginal bleeding (67.76%), the most common sign was uterine enlargement (46.75%).

As the ratio of caesarean scar pregnancy increases, the caesarean delivery rate should be decreased to decrease the morbidity of caesarean scar pregnancy.

## Background

Ectopic pregnancy occurs when a fertilized ovum implants outside the endometrium of the uterine cavity, which is a life-threatening occurrence and is an important cause of pregnancy-related mortality [1]. Tubal pregnancy is the most common type of ectopic pregnancy. Uncommon types of ectopic pregnancy include ovarian pregnancy, cervical pregnancy, abdominal pregnancy, and caesarean scar pregnancy.

The site of ectopic pregnancy can affect the clinical syndrome, treatment and prognosis of patients. For example, ovarian pregnancy is easier to rupture compared with tubal pregnancy [2], which means ovarian pregnancy needs more active management. Caesarean scar pregnancy has the possibility of massive haemorrhage and uterine rupture [3]. It is important to identify the site of ectopic pregnancy when we encounter a patient who may have an ectopic pregnancy, to generate different treatment strategy for different ectopic pregnancy, but the distribution of ectopic pregnancy sites has been insufficiently studied.

With the increase in pelvic and intrauterine operations, the distribution of ectopic sites has been changing, but the variation has been insufficiently studied. To investigate the distribution of ectopic sites and its variation trend in depth, we collected the data on ectopic pregnancy cases from the Third Affiliated Hospital of Sun Yat-Sen University to analyse the variation trend of ectopic sites.

## Materials and methods

This was a retrospective descriptive study. We analysed the data of ectopic pregnancy patients from 2011 to 2019 from the Gynecology Department of the Third Affiliated Hospital of Sun Yat-Sen University. From 2011 to 2019, 3915 ectopic pregnancy patients were hospitalized. The sites of ectopic pregnancy were determined by the operating surgeons and pathologists or by ultrasound. The following sites were included in our study: interstitial pregnancy, isthmic pregnancy, ampullary pregnancy, fimbrial pregnancy, ovarian pregnancy, abdominal pregnancy, cervical pregnancy, caesarean scar pregnancy, cornual pregnancy and heterotopic pregnancy.

After calculating the distribution of the implantation site of ectopic pregnancy, we compared the trend of the distribution of ectopic pregnancy sites through χ^2^ tests for trend, classified cases into the years 2012–2015 and 2016–2019, and analysed the variation trend through χ^2^ tests. All statistical analyses were carried out with SPSS version 25.

## Results

### The sites of ectopic pregnancy

Tubal pregnancy accounted for 84.70% of ectopic pregnancies. Because some cases were diagnosed by ultrasound tests, or because the ectopic mass ruptured or aborted, which leads to the difficulty in confirming the ectopic site, we could not determine the ectopic site of 953 tubal pregnancy cases. Ampullary pregnancy constituted 89.21% of tubal pregnancies (Tables 1, 2).

**Table 1:** The ectopic site distribution of 3915 ectopic pregnancy cases

Site	Tubal pregnancy (n)	Ovarian pregnancy (n)	Caesarean scar pregnancy (n)	Abdominal pregnancy (n)	Cornual pregnancy (n)	Cervical pregnancy (n)	Heterotopic pregnancy (n)	Other sites	Total
n	3316	61	338	24	105	19	17	35	3915
%	84.70	1.56	8.63	0.61	2.68	0.49	0.43	0.89

**Table 2:** Distribution of the sites of tubal pregnancy

Site	Interstitial pregnancy	Isthmic pregnancy	Ampullary pregnancy	Fimbrial pregnancy	Total
n	80	114	2108	61	2363
%	3.39	4.82	89.21	2.58	

### The variations of sites of ectopic pregnancy

The site of ectopic pregnancy was calculated year by year. Because the branch hospital began operations in 2011, the data from 2011 were not included (Table 3).

**Table 3:** The ectopic site distribution for each year

Year	Tubal pregnancy (n)	Ovarian pregnancy (n)	Caesarean scar pregnancy (n)	Abdominal pregnancy (n)
2012	372 (91.62%)	3 (0.74%)	22 (5.42%)	5 (1.23%)
2013	383 (93.41%)	7 (1.71%)	16 (3.90%)	0 (0.00%)
2014	420 (88.61%)	6 (1.27%)	31 (6.54%)	1 (0.21%)
2015	347 (86.75%)	9 (2.25%)	28 (7.00%)	2 (0.50%)
2016	414 (81.02%)	6 (1.17%)	67 (13.11%)	2 (0.39%)
2017	434 (82.51%)	5 (0.95%)	52 (9.89%)	7 (1.33%)
2018	397 (80.86%)	9 (1.83%)	59 (12.02%)	0 (0.00%)
2019	339 (79.21%)	8 (1.87%)	53 (12.38%)	6 (1.40%)
*P*	< 0.001	0.704	0.005	0.826

Year	Cornual pregnancy (n)	Cervical pregnancy (n)	Heterotopic pregnancy (n)	Total
2012	3 (0.74%)	0 (0.00%)	1 (0.25%)	406
2013	4 (0.98%)	0 (0.00%)	0 (0.00%)	410
2014	15 (3.16%)	1 (0.21%)	0 (0.00%)	474
2015	10 (2.50%)	3 (0.75%)	1 (0.25%)	400
2016	13 (2.54%)	5 (0.98%)	4 (0.78%)	511
2017	19 (3.61%)	4 (0.76%)	5 (0.95%)	526
2018	18 (3.67%)	3 (0.61%)	5 (1.02%)	491
2019	20 (4.67%)	1 (0.23%)	1 (0.23%)	428
*P*	0.037	0.382	0.256	

The proportion of tubal pregnancy showed a downward trend, and the proportions of caesarean scar pregnancy and cornual pregnancy showed an upward trend.

As the cases of different ectopic pregnancy showed a fluctuating trend, to better compare the trend of the distribution of ectopic pregnancy sites, we divided the cases into the years 2012–2015 and 2016–2019 (Table 4).

**Table 4:** The comparison of distribution sites for 2012–2015 and 2016–2019

Year	Tubal pregnancy (n)	Ovarian pregnancy (n)	Caesarean scar pregnancy (n)	Abdominal pregnancy (n)
2012–2015	1522 (90.06%)	25 (1.48%)	97 (5.74%)	8 (0.47%)
2016–2019	1584 (80.98%)	28 (1.43%)	231 (11.81%)	15 (0.77%)
*P*	< 0.001	0.506	< 0.001	0.183

Year	Cornual pregnancy (n)	Cervical pregnancy (n)	Heterotopic pregnancy (n)	Total
2012–2015	32 (1.89%)	4 (0.24%)	2 (0.12%)	1690
2016–2019	70 (3.58%)	13 (0.66%)	15 (0.77%)	1956
*P*	< 0.001	0.046	0.003	

From 2012 to 2015 and 2016–2019, the proportion of tubal pregnancy decreased from 90.06 to 80.98% (*P* < 0.001), and the ratio of caesarean scar pregnancy to ectopic pregnancy increased from 5.74 to 11.81% (*P* < 0.001). The ratios of cornual pregnancy, cervical pregnancy and heterotopic pregnancy to ectopic pregnancy all showed an upward trend, but there was only a small number of cornual pregnancy, cervical pregnancy and heterotopic pregnancy cases.

### The clinical characteristics of caesarean scar pregnancy

As the ratio of caesarean scar pregnancy to ectopic pregnancy showed an upward trend, the clinical data of caesarean scar pregnancy was collected and analysed (Table 5).

**Table 5:** The clinical characteristics of caesarean scar pregnancy

	n = 338	%
Age (y)		
< 20	0	0.00
20–29	81	23.96
30–39	227	67.16
≥ 40	30	8.88
Mean ± SD	32.90 ± 4.80	
Gestational age (weeks)		
< 6	74	23.57
6 ~ 8	167	53.18
≥ 8	73	23.25
Unclear gestational age	24	
Mean ± SD	6.67 ± 1.82	
Gestation history		
Childbearing history	338	100.00
Abortion history	271	80.18
Ectopic pregnancy history	22	6.51
Pelvic disease history	3	0.89
Pelvic surgery history (except caesarean delivery)	28	8.28
Caesarean delivery history		
1	246	72.78
2	85	25.15
3	7	2.07
Assisted reproduction history	3	0.89
Birth control history	8	2.37
Intrauterine contraceptive device	2	0.59
Oral contraceptive	5	0.15
Ligation operation	1	0.30
Clinical manifestations		
Amenorrhea	333	98.52
Abdominal pain	87	25.74
Vaginal bleeding	229	67.76
Syncope	0	0.00
Shock	0	0.00
Signs		
Adnexal mass	6	1.78
Adnexal tenderness	4	1.18
Cervical pain when lifting	9	2.66
Uterine enlargement	158	46.75
Aspirating blood during culdocentesis	0	0.00
HCG (U/L)		
HCG < 20,000	100	39.06
20,000 ≤ HCG < 100,000	110	42.97
HCG ≥ 100,000	46	17.97
Unclear HCG	112	
Progesterone (nmol/L)		
P < 60	134	56.07
60 ≤ P < 120	93	38.91
P ≥ 120	12	5.02
Unclear P	129	
Treatment methods		
Suction curettage	136	40.23
Suction curettage + uterine artery embolization	126	37.28
Other surgical treatment methods	54	15.98
Conservative treatment methods	6	1.78
Refused treatments	16	4.73

The mean age was 32.90 ± 4.80 years old, and the mean gestational age was 6.67 ± 1.82 weeks. A total of 72.78% (246/338) of patients had one caesarean delivery, 25.15% (85/338) had two caesarean deliveries, and 2.07% (7/338) had three caesarean deliveries. The most common clinical manifestations were amenorrhea, abdominal pain and vaginal bleeding, and the most common sign was uterine enlargement. HCG was almost at the level of 20,000–100,000 U/L, and progesterone was almost less than 60 nmol/L. A total of 40.23% (126/338) of patients were treated by suction curettage, and 37.28% (126/338) of patients were treated by suction curettage + uterine artery embolization. Patients were also treated by conservative treatment methods, such as hysteroscopy and laparoscopy.

## Discussion

The site of ectopic pregnancy can affect the clinical syndrome of patients. Compared with tubal pregnancy, ovarian pregnancy ruptures more easily, which leads to a higher shock rate and requires more emergency management [2]. The mortality rate of abdominal pregnancy is eight times higher than that of tubal pregnancy, with a mortality rate of 0.5–18% for late diagnosis and treatment [4, 5]. Having a clear picture of the distribution of ectopic pregnancy can help us better make clinical decisions when we encounter a patient with an ectopic pregnancy.

However, the site of ectopic pregnancy has been insufficiently studied. Most studies focus on the trend of the ectopic pregnancy ratio of all female populations or focus on the mortality rate of one specific type of ectopic pregnancy. Tubal pregnancy is the most common type of ectopic pregnancy. According to previous studies, approximately 95% of ectopic pregnancy is tubal pregnancy [6], ovarian pregnancy makes up approximately 0.5%-3% of ectopic pregnancy with an incidence rate of 1/7000–1/40,000 live births [7–9], abdominal pregnancy makes up about 1.3% of ectopic pregnancy with an incidence rate ranging from 1 in 10,000 to 30,000 pregnancies [10, 11], cervical pregnancy comprises about 1% of ectopic pregnancy with an incidence rate varying between 1 in 2,500 and 1 in 12,422 pregnancies [12, 13], and caesarean scar pregnancy makes up approximately 6% of ectopic pregnancy with an incidence rate of approximately 1 in 2500 to 1 in 1800 of pregnancies [14, 15]. Most studies only focus on a specific type of ectopic pregnancy, and we can only obtain general information about the ectopic pregnancy distribution. According to our data, tubal pregnancy consists of 84.70% of ectopic pregnancy cases, which is lower than the 95% reported in previous studies. The proportions of ovarian pregnancy, abdominal pregnancy, cervical pregnancy, caesarean scar pregnancy and cornual pregnancy are close to those in previous studies.

According to the study by Bouyer [16] in 2001, interstitial pregnancy accounted for 2.4% of ectopic pregnancy, isthmic pregnancy consisted of 12%, ampullary pregnancy accounted for 70%, fimbrial pregnancy comprised 11.1%, ovarian pregnancy accounted for 3.2% and abdominal pregnancy comprised 1.3%. According to our data, interstitial pregnancy consisted of 3.39%, isthmic pregnancy consisted of 4.82%, ampullary pregnancy accounted for 89.21%, and fimbrial pregnancy accounted for 2.58%.

With the increase in the caesarean delivery rate in China, the incidence rate of caesarean scar pregnancy has been increasing in recent years. Caesarean scar pregnancy is a special type of ectopic pregnancy in which embryos are implanted at caesarean scar. Caesarean scar pregnancy can lead to severe complications, such as severe haemorrhage and uterine rupture [17]. According to the study by Li Hong-Tian [18], during the years 2008–2018, the caesarean delivery rate increased from 28.8 to 36.7%.

The proportion of tubal pregnancy showed a downward trend, the proportions of caesarean scar pregnancy and cornual pregnancy showed an upward trend. From 2012 to 2015 and 2016–2019, the proportion of caesarean scar pregnancy increased from 5.74 to 11.81%, which reminds us that the caesarean delivery rate should be decreased to decrease the morbidity of caesarean scar pregnancy.

From 2012 to 2015 and 2016–2019, the ratio of tubal pregnancy to ectopic pregnancy decreased from 90.06 to 80.98%. This may be because of the rise in women’s health awareness, which leads to a decrease in the rate of pelvic inflammatory disease and tubal diseases. According to the study by Kreisel [19], the ratio of emergency department visits due to pelvic inflammatory disease decreased from 0.57% in 2006 to 0.41% in 2013. In addition, patients are more willing to treat tubal infertility, which also leads to the decrease in tubal diseases.

The ratio of cornual pregnancy to ectopic pregnancy increased from 1.89 to 3.58%, which may be related to the increase in intrauterine operations and the damage to the endometrium due to those operations. It’s important to decrease the number of unnecessary intrauterine operations.

Caesarean scar pregnancy means a fertilized ovum implants at the caesarean scar. After implantation, trophoblasts can invade the myometrium and grow there, which may lead to uterine rupture or massive bleeding. If it keeps growing, caesarean scar pregnancy can develop into placenta previa, placenta implantation and dangerous placenta previa, bringing great risks to pregnant women [20]. Placenta previa, placenta implantation and dangerous placenta previa may lead to massive haemorrhage, infection, premature delivery, fetal asphyxia, shock and death. Blood transfusion, caesarean, and even hysterorrhexis may be needed to save lives.

Caesarean scar pregnancy is highly associated with a caesarean delivery history, but the pathogenesis is still unclear; its pathogenesis may be the broadening of the scar, fibrosis and ischaemia of the uterine wall, and poor healing of the scar [21]. According to previous studies, high-risk factors for caesarean scar pregnancy are abortion history, multiple caesarean delivery history, suture method and the intervals between caesarean deliveries [22]. The scars of patients with double-layer sutures are thicker than those of patients with one-layer sutures [23]. This is in accordance with the data at our hospital. A total of 72.78% (246/338) of patients had one caesarean delivery, 25.15% (85/338) had two caesarean deliveries, and 2.07% (7/338) had three caesarean deliveries. A total of 80.18% (271/338) had aborted before.

According to the study by So Yun Kim [24], the mean age at which caesarean scar pregnancy occurs is 35.7 ± 3.8 years old, the mean gestational age at diagnosis is 6.5 ± 1.1 weeks and the mean hCG level before treatment is 30,785 (range 550–155,356) U/L. According to Lanrong Luo [25], the mean age of individuals with caesarean scar pregnancy is 34.16 ± 4.4 years old. According to our data, the mean age was 32.90 ± 4.80 years old, 67.16% (227/338) of patients were between 30 and 39 years old, and the mean gestational age was 6.67 ± 1.82 weeks, which is similar to previous studies.

The diagnosis of caesarean scar pregnancy is mainly through ultrasound tests, especially transvaginal ultrasound combined with transabdominal ultrasound, Magnetic Resonance Imaging can be used to clarify the relationship between the gestation sac and other organs when necessary. The main clinical manifestations of caesarean scar pregnancy are amenorrhea, abdominal pain and vaginal bleeding [26], similar to other kinds of ectopic pregnancies. According to our data, the most common clinical manifestations are amenorrhea (98.52%), abdominal pain (25.74%) and vaginal bleeding (67.76%), and the most common sign is uterine enlargement (46.75%).

The main treatment methods for caesarean scar pregnancy are suction curettage, suction curettage + uterine artery embolization, hysteroscopy, hysteroscopy + uterine artery embolization and laparoscopy. Uterine artery embolization can greatly reduce the possibility of massive haemorrhage. It can also be treated by conservative treatment methods, using localized or systematic methotrexate [26]. At our hospital, 40.23% (126/338) of patients were treated by suction curettage, and 37.28% (126/338) of patients were treated by suction curettage + uterine artery embolization. Suction curettage and suction curettage + uterine arterial embolization are the dominant treatment methods.

## Conclusions

Tubal pregnancy makes up 84.70% of ectopic pregnancy, which is lower than the previous data. Caesarean scar pregnancy accounts for 8.63% of ectopic pregnancy. The proportion of caesarean scar pregnancy showed an upward trend. As caesarean scar pregnancy has the possibility of massive haemorrhage and hysterorrhexis, the caesarean delivery rate should be decreased to decrease the morbidity of caesarean scar pregnancy.

Caesarean scar pregnancy is highly associated with a history of caesarean delivery. The most common clinical manifestations are amenorrhea, abdominal pain and vaginal bleeding, and the most common sign is uterine enlargement. Caesarean scar pregnancy can be treated by different methods, and suction curettage and suction curettage + uterine artery embolization are the dominant treatment methods at our hospital.

## Data Availability

The datasets used and analyzed during the current study are available from the corresponding author on reasonable request.
